# Supplementary material for: Lefamulin in Patients with Community-Acquired Bacterial Pneumonia Caused by Atypical Respiratory Pathogens: Pooled Results from Two Phase 3 Trials
Source: Antibiotics (Basel). 2021 Dec 4;10(12):1489. doi: 10.3390/antibiotics10121489 (PMC8698636; doi:10.3390/antibiotics10121489)
Supplement: Supplementary file 1 [file antibiotics-10-01489-s001.zip › antibiotics-1426730-supplementary.pdf]

Supplementary Materials

Table S1. Patient Demographic and Baseline Characteristics by Atypical Pathogen*

	**Patients With *M. pneumoniae* (Pooled microITT Population)**		**Patients With *L. pneumophila* (Pooled microITT Population)**		**Patients With *C. pneumoniae* (Pooled microITT Population)**	
**Parameter**	**Lefamulin (*n*=39)**	**Moxifloxacin (*n*=34)**	**Lefamulin (*n*=34)**	**Moxifloxacin (*n*=31)**	**Lefamulin (*n*=27)**	**Moxifloxacin (*n*=31)**
Age, y, median (range)	54 (19–87)	52 (19–92)	60 (25–89)	61 (26–89)	59 (25–83)	64 (20–92)
Age ≥65 y, *n* (%)	11 (28.2)	12 (35.3)	11 (32.4)	12 (38.7)	7 (25.9)	15 (48.4)
Male, *n* (%)	16 (41.0)	18 (52.9)	25 (73.5)	18 (58.1)	19 (70.4)	18 (58.1)
White, *n* (%)	34 (87.2)	28 (82.4)	31 (91.2)	28 (90.3)	27 (100)	27 (87.1)
PORT risk class, *n* (%)						
I/II[†]	15 (38.5)	8 (23.5)	7 (20.6)	7 (22.6)	8 (29.6)	6 (19.4)
III	19 (48.7)	19 (55.9)	19 (55.9)	16 (51.6)	16 (59.3)	12 (38.7)
IV/V[†]	5 (12.8)	7 (20.6)	8 (23.5)	8 (25.8)	3 (11.1)	13 (41.9)
CURB-65 score,[‡] *n* (%)						
0–2	37 (94.9)	31 (91.2)	32 (94.1)	29 (93.5)	27 (100)	28 (90.3)
3–5	2 (5.1)	3 (8.8)	2 (5.9)	2 (6.5)	0	3 (9.7)
Minor ATS severity criteria,[§] *n* (%)	7 (17.9)	6 (17.6)	7 (20.6)	4 (12.9)	1 (3.7)	3 (9.7)
Modified ATS severity criteria,[‖] *n* (%)	3 (7.7)	3 (8.8)	5 (14.7)	4 (12.9)	0	3 (9.7)
Multilobar pneumonia, *n* (%)	8 (20.5)	8 (23.5)	9 (26.5)	6 (19.4)	3 (11.1)	6 (19.4)
SIRS,[¶] *n* (%)	39 (100)	32 (94.1)	33 (97.1)	27 (87.1)	26 (96.3)	30 (96.8)
Bacteremic, *n* (%)	0	0	0	0	0	1 (3.2)
Prior antibiotic use,[#] *n* (%)	14 (35.9)	8 (23.5)	10 (29.4)	8 (25.8)	7 (25.9)	8 (25.8)
Renal status,[**] *n* (%)						
Normal	27 (69.2)	21 (61.8)	19 (55.9)	14 (45.2)	16 (59.3)	15 (48.4)
Mild impairment	6 (15.4)	8 (23.5)	12 (35.3)	10 (32.3)	10 (37.0)	10 (32.3)

Moderate impairment	5 (12.8)	5 (14.7)	3 (8.8)	7 (22.6)	0	6 (19.4)
Severe impairment	1 (2.6)	0	0	0	1 (3.7)	0
Missing	0	0	0	0	0	0
Medical history,[††] *n* (%)						
Smoking history	12 (30.8)	9 (26.5)	18 (52.9)	10 (32.3)	9 (33.3)	8 (25.8)
Hypertension	12 (30.8)	10 (29.4)	16 (47.1)	11 (35.5)	9 (33.3)	12 (38.7)
Asthma/COPD	1 (2.6)	4 (11.8)	8 (23.5)	3 (9.7)	1 (3.7)	5 (16.1)
Diabetes mellitus	0	4 (11.8)	5 (14.7)	6 (19.4)	3 (11.1)	4 (12.9)
Baseline pathogen, n (%)[‡‡]						
Mycoplasma pneumoniae	39 (100)	34 (100)	4 (11.8)	3 (9.7)	2 (7.4)	4 (12.9)
Legionella pneumophila	4 (10.3)	3 (8.8)	34 (100)	31 (100)	4 (14.8)	4 (12.9)
Chlamydia pneumoniae	2 (5.1)	4 (11.8)	4 (11.8)	4 (12.9)	27 (100)	31 (100)
Streptococcus pneumoniae	12 (30.8)	10 (29.4)	6 (17.6)	10 (32.3)	7 (25.9)	11 (35.5)
Haemophilus influenzae	4 (10.3)	6 (17.6)	3 (8.8)	3 (9.7)	1 (3.7)	4 (12.9)
Moraxella catarrhalis	4 (10.3)	1 (2.9)	2 (5.9)	0	1 (3.7)	0
Staphylococcus aureus	2 (5.1)	0	0	1 (3.2)	3 (11.1)	0

ATS, American Thoracic Society; BUN, blood urea nitrogen; CABP, community-acquired bacterial pneumonia; COPD, chronic obstructive pulmonary disease; CrCl, creatinine clearance; HLT, high-level term; ITT, intent to treat; MedDRA, Medical Dictionary for Regulatory Activities; microITT, microbiological ITT; NEC, not elsewhere classified; PORT, Pneumonia Outcomes Research Team; SIRS, systemic inflammatory response syndrome; WBC, white blood cell (count).

*Defined as *M. pneumoniae*, *L. pneumophila*, and *C. pneumoniae*.

[†]PORT risk class I/II and IV/V for the pooled ITT population but PORT risk class II and IV, respectively, for patients with atypical pathogens. PORT risk class calculated programmatically using site data reported in the electronic case report form was not always consistent with the site-reported PORT risk class used for enrollment/stratification.

[‡]Defined as confusion of new onset, BUN >19 mg/dL, respiratory rate ≥30 breaths/min, systolic blood pressure <90 mm Hg or diastolic blood pressure ≤60 mm Hg, and age ≥65 years.

[§]Defined as baseline presence of ≥3 of the following 9 criteria: respiratory rate ≥30 breaths/min, O_2 saturation <90% or PaO_2 <60 mm Hg, BUN ≥20 mg/dL, WBC <4000 cells/mm^3, confusion, multilobar infiltrates, platelets <100,000 cells/mm^3, temperature <36°C, or systolic blood pressure <90 mm Hg [17].

[||]Defined as baseline presence of ≥3 of the following 6 criteria: respiratory rate ≥30 breaths/min, SpO_2/FiO_2 <274 where SpO_2/FiO_2 = 64+0.84 (PaO_2/FiO_2), BUN ≥20 mg/dL, confusion, age ≥65 years, or multilobar infiltrates [62].

[¶]Defined as baseline presence of ≥2 of the following 4 criteria: temperature <36°C or >38°C; heart rate >90 bpm; respiratory rate >20 breaths/min; and WBC <4000 cells/mm^3, WBC >12,000 cells/mm^3, or immature polymorphonuclear neutrophils >10%.

[#]Patients received a single dose of short-acting systemic antibacterial medication ≥72 hours before randomization; randomization was stratified and capped such that ≥25% of the total ITT population met these criteria.

[**]Defined as normal (CrCl ≥90 mL/min), mild (CrCl 60–<90 mL/min), moderate (CrCl 30–<60 mL/min), and severe (CrCl <30 mL/min).

[††]Medical history terms were defined as follows: hypertension = MedDRA HLT "vascular hypertensive disorders NEC"; asthma/COPD = MedDRA HLT "bronchospasm and obstruction"; diabetes mellitus = MedDRA HLT "diabetes mellitus (incl subtypes)."

[‡‡]Among the subpopulation of patients with atypical pathogens (*M. pneumoniae, L. pneumophila, C. pneumoniae*), all patients had ≥1 atypical pathogen at baseline, with the corresponding infections being either mono- or polymicrobial. Within those polymicrobial infections that occurred in patients with atypical pathogens, additional baseline pathogens of *S. pneumoniae*, *H. influenzae, M. catarrhalis,* and *S. aureus* were identified.

Table S2. Overall Summary of TEAEs by Atypical Pathogen*

Patients, *n* (%)	Patients With *M. pneumoniae* (Pooled microITT Population) Lefamulin (*n*=39)	Patients With *M. pneumoniae* (Pooled microITT Population) Moxifloxacin (*n*=34)	Patients With *L. pneumophila* (Pooled microITT Population) Lefamulin (*n*=34)	Patients With *L. pneumophila* (Pooled microITT Population) Moxifloxacin (*n*=31)	Patients With *C. pneumoniae* (Pooled microITT Population) Lefamulin (*n*=27)	Patients With *C. pneumoniae* (Pooled microITT Population) Moxifloxacin (*n*=31)
Any TEAE†	16 (41.0)	9 (26.5)	11 (32.4)	10 (32.3)	7 (25.9)	10 (32.3)
Mild	10 (25.6)	7 (20.6)	5 (14.7)	4 (12.9)	2 (7.4)	6 (19.4)
Moderate	5 (12.8)	2 (5.9)	5 (14.7)	3 (9.7)	3 (11.1)	2 (6.5)
Severe	1 (2.6)	0	1 (2.9)	3 (9.7)	2 (7.4)	2 (6.5)
Related TEAE‡	6 (15.4)	3 (8.8)	1 (2.9)	2 (6.5)	1 (3.7)	2 (6.5)
Serious TEAE	1 (2.6)	1 (2.9)	3 (8.8)	2 (6.5)	3 (11.1)	2 (6.5)
Related serious TEAE	0	0	0	0	0	0
TEAE leading to study drug discontinuation	0	0	0	2 (6.5)	0	2 (6.5)
TEAE leading to death (over entire study duration)	1 (2.6)§	0	0	0	0	0
28-d all-cause mortality – deceased at Day 28‖	0	0	0	0	0	0
TEAEs by SOC in ≥5% of patients in any treatment group¶						
Gastrointestinal disorders	4 (10.3)	4 (11.8)	1 (2.9)	2 (6.5)	2 (7.4)	1 (3.2)
Infections and infestations	3 (7.7)	2 (5.9)	2 (5.9)	1 (3.2)	2 (7.4)	4 (12.9)
Investigations	2 (5.1)	1 (2.9)	1 (2.9)	2 (6.5)	2 (7.4)	1 (3.2)
Blood and lymphatic system disorders	2 (5.1)	0	2 (5.9)	1 (3.2)	0	0
General disorders and administration site conditions	2 (5.1)	0	2 (5.9)	0	1 (3.7)	0
Cardiac disorders	1 (2.6)	2 (5.9)	0	0	0	0
Respiratory, thoracic, and mediastinal disorders	0	0	4 (11.8)	1 (3.2)	2 (7.4)	0
Vascular disorders	0	0	0	2 (6.5)	0	0
Renal and urinary disorders	0	0	0	0	0	2 (6.5)

COPD, chronic obstructive pulmonary disease; MedDRA, Medical Dictionary for Regulatory Activities; microITT, microbiological intent to treat; PORT, Pneumonia Outcomes Research Team; PT, preferred term; SOC, system organ class; TEAE, treatment-emergent adverse event.
[*]Defined as *M. pneumoniae*, *L. pneumophila*, and *C. pneumoniae*.
[†]TEAEs started or worsened during or after first study drug administration (an adverse event with an unknown start date or partial date was categorized as a TEAE); patients with multiple events in a given category were only counted once.
[‡]TEAEs that were "Definitely," "Probably," or "Possibly" related to the study drug. If the TEAE relationship was missing, it was treated as "Related."
[§]One patient (aged 70 years; PORT risk class II; moderate renal impairment [creatinine clearance 30 to <60 mL/min] at baseline; history of hypertension and COPD; baseline pathogens *Haemophilus influenzae*, *Haemophilus parainfluenzae*, and *Mycoplasma pneumoniae*) in the lefamulin group had a TEAE leading to death after study day 28, the patient died on study day 271 from acute myeloid leukemia (first reported on study day 269).
[||]Assessed in the intent-to-treat population (lefamulin *n*=646; moxifloxacin *n*=643).
[¶]Although a patient may have had >1 TEAE, the patient was counted only once within an SOC category and once within a PT category. The same patient may have contributed ≥2 PTs in the same SOC category, but the patient was only counted once towards that SOC category. Adverse events were coded using MedDRA version 20.0.
